# Air Pollution, Socioeconomic Status, and Children's Cognition in Megacities: The Mexico City Scenario

**DOI:** 10.3389/fpsyg.2012.00217

**Published:** 2012-07-09

**Authors:** Lilian Calderón-Garcidueñas, Ricardo Torres-Jardón

**Affiliations:** ^1^Instituto Nacional de PediatríaMexico City, Mexico; ^2^The Center for Structural and Functional Neurosciences, The University of MontanaMissoula, MT, USA; ^3^Centro de Ciencias de la Atmósfera, Universidad Nacional Autónoma de MéxicoMexico City, Mexico

## Introduction

**Figure 1:**
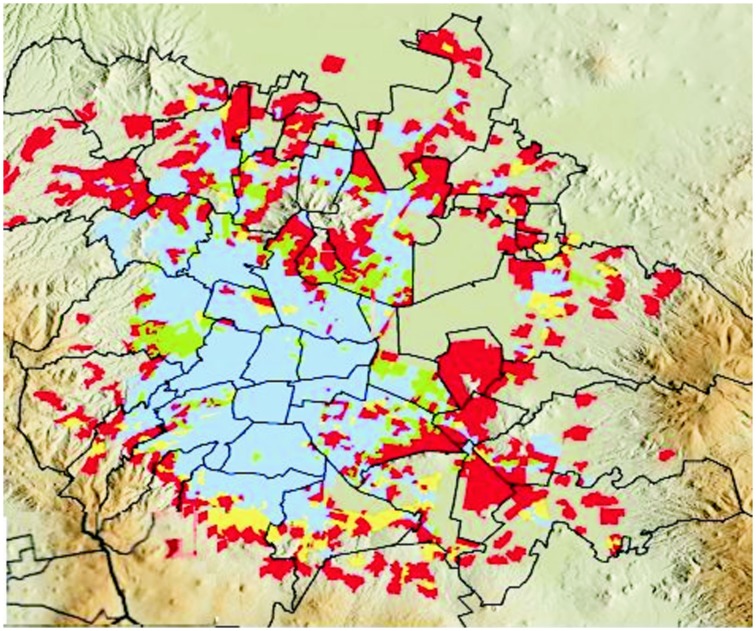
**Location of vulnerable population and housing groups in MC**. Blue: not vulnerable; Green: vulnerable population; Yellow: vulnerable housing; Red: vulnerable population and housing. Population vulnerable areas have high concentration of people >18 years without secondary education and low SES. Housing vulnerable groups live in areas of poor construction housing, without access to basic services, and limited property ownership/rights. The black line shows the limits of the Federal District. (Figure reproduced with permission from The World Bank, 2011).

Megacities around the world have significant problems with air pollution (Molina and Molina, 2004; Chen and Kan, 2008; Parrish and Zhu, 2009). Metropolitan Mexico City (MC) – an example of extreme urban growth and serious environmental pollution with 20 million people, over 40,000 industries and four million vehicles – exhibits marked regional differences in air pollutants concentrations including industrial and mobile sources of contaminants (Ezcurra and Mazari-Hiriart, 1996; Moreno et al., 2008; Querol et al., 2008; Salcedo et al., 2010). Eight million MC children experienced serious detrimental effects including neuroinflammation, neurodegeneration, and cognition deficits (Calderón-Garcidueñas et al., 2008a,b,c, 2011a,b, 2012). Neighborhoods’ proximity to main roadways, unpaved roads, dumps, and factories affect children's health and 27% of the MC population mostly to the north and east falls in this category (Figure 1).

While both children living in North Mexico City (NMC) and those in South Mexico City (SMC) are exposed to air pollution, the specific pollutant agents are quite different. NMC children are exposed to high concentrations of fine particulate matter (PM_2.5_), and its constituents: organic and elemental carbon, secondary inorganic aerosols and metals (Zn, Cu, Pb, Ti, Mn, Sn, V, Ba), while SMC children are exposed to high ozone concentrations and PM-associated with lipopolysaccharides. This is important because exposure to specific pollutants may lead to specific detrimental effects (Rivas-Arancibia et al., 2010; Villarreal-Calderon et al., 2010; Levesque et al., 2011).

The neuropathology in MC mongrel dogs have shown DNA oxidative damage, Alzheimer's-type pathology, and accumulation of combustion-associated metals in olfactory mucosa, olfactory bulb, and frontal cortex, suggesting Alzheimer-like pathology and the resulting systemic and brain inflammation could be the consequence of air pollutant exposures (Calderón-Garcidueñas et al., 2002, 2003). In humans, the extensive respiratory inflammation targets the nasal epithelium first (Calderón-Garcidueñas et al., 1992). The pulmonary damage is equally severe and boys are more affected than girls, an observation likely related to their longer outdoor exposure hours (Calderón-Garcidueñas et al., 2003). Systemic inflammation, endothelial dysfunction, and high concentrations of interleukin-1β and tumor necrosis factor-alpha (TNF-α) with major impact in the brain (endothelial cells have receptors for these inflammatory mediators) are the norm in exposed children. These children exhibit high concentrations of endothelin-1, a potent vasoconstrictor that impacts the brain microvasculature (Calderón-Garcidueñas et al., 2008a). Therefore, it came as no surprise to see the pathological, structural, and cognitive effects of the MC exposures in otherwise clinically healthy children (Calderón-Garcidueñas et al., 2011a). SMC children carefully selected for unremarkable clinical histories and no known risk factors for neurological or cognitive disorders compared to clean-air-controls matched for age, gender, and socioeconomic status (SES), exhibited significant deficits in a combination of fluid and crystallized cognition tasks (Calderón-Garcidueñas et al., 2008b), and 56% of MC children exhibited prefrontal white matter hyperintensities (WMH) similar to those in young dogs (57%). Even more striking, their cognitive deficits matched their MRI volumetric changes in their right parietal and bilateral temporal areas. Thus, exposure to air pollution may perturb the trajectory of cerebral development and result in cognitive deficits during childhood (Calderón-Garcidueñas et al., 2011a).

The MC children's neuropathology explain some of the clinical, electrophysiological, and brain MRI findings in our cohorts. The delayed brainstem auditory evoked potentials for example, correlate with the accumulation of α-synuclein and/or beta amyloid in auditory and vestibular nuclei (Kulesza and Muguray, 2008; Calderón-Garcidueñas et al., 2011b). In an autopsy cohort of 43 children and young adults (35 MC and 8 CTL), 40% exhibited frontal tau hyperphosphorylation with pre-tangle material and 51% had amyloid diffuse plaques compared with 0% in controls (Calderón-Garcidueñas et al., 2012). Hyperphosphorylated tau and amyloid plaques are seen in Alzheimer's disease and the development of neurodegenerative diseases must be contemplated as a potential long-term effect in exposed children.

It is clear that MC children, regardless of SES, are not healthy and detrimental short-term brain effects and potentially serious long-term effects are expected. Thus, in addressing the early cognitive and brain structural detrimental effects, we ask: do MC children have the capacity to recover from the observed negative neurological effects?

Anderson et al. (2011) reviewed how both early plasticity and early vulnerability may reflect opposite extremes along a “recovery *continuum*” which, we argue, is pertinent to our children. The detrimental pollution effects likely start *in utero* and continue relentlessly as the child grows up. Children's brains are fully capable of plasticity and neural compensation, thus our MRI observations of increased white matter volume (Calderón-Garcidueñas et al., 2011a), in connection with a well defined vascular lesion associated with low blood flow (Foscarin et al., 2012), is not surprising. Neural compensation has been described in association with white matter lesions, infarcts, and in healthy subjects as a function of training and experience. Specifically, Duffau (2009) compensatory mechanisms following white matter damage included: unmasking of peri-lesional latent networks, recruitment of accessory pathways, introduction of additional relays within the circuit, and involvement of parallel long-distance association pathways. If the child's responses to a single insult depend on a complex set of factors (the nature of the insult, the severity, the timing, cognitive reserve, genetic makeup, nutrition status, family function, etc.), the responses of a child continuously exposed to a polluted environment may even be more complex. Her capacity to compensate and overcome the developmental disruption may be far more intricate given the neuroinflammation and the presence of pathological markers of neurodegeneration (Calderón-Garcidueñas et al., 2012).

## Why are Low SES Children More Vulnerable than Middle or High SES Children in MC?

Low SES children lack the support they need to develop what Diamond and Lee (2011) consider the four qualities required to be successful: creativity, flexibility, self-control, and discipline. Accordingly, low SES children in MC attend public schools that are well known for deficient curricular programs, lack of creativity, flexibility or disciplinary practices, and teachers’ unprofessionalism and absenteeism (Loret de Mola and García Bernal, 2012). Deficient schools do not help in the development of executive function skills and do not build cognitive reserves. Low SES children in MC have emotional and social needs, which are also exacerbated by domestic, school, and street violence (Cicchetti et al., 2010; Liu, 2011). These children are facing a significant rise in crime and violence in their neighborhoods (Davis, 2009) in a country where 52 million people have incomes below the poverty line.

On the other hand, typically children from middle and high SES families have access to balanced nutrition and cognitively stimulating home and school environments. Have parents that can afford services and resources for their specific need, and attend schools with stimulating curricula, including teaching a second language. Middle or high SES children live in neighborhoods with lower crime rate and have access to private pediatric care. Thus, the factors accounting for chronic stress in low SES children are typically not present in the higher income cohorts. In keeping with Cicchetti et al. (2010, 2011), Rogosch et al. (2011), and Sturge-Apple et al. (2012) the significant impact of high environmental stress is likely affecting predominantly low SES MC children.

## Conclusion

Do we all breathe the same air in MC? Not quite. Low SES children are more likely to be live in environmental unjust communities, are exposed to second and third hand tobacco smog, and are more likely smokers themselves. They have higher chances of residing in high-density multiunit dwellings, with proximity to high traffic streets and factories, gas stations, mechanical shops, or share their living spaces with a home polluting business.

Environmental justice/inequity studies suggest the level of pollution present in the environment in which vulnerable populations reside is higher than in more affluent areas (Jerrett et al., 2001; Morello-Frosch et al., 2002; Prochaska et al., 2012). Subjects in poor areas are more likely to spend time close to or in traffic, working on the street, walking long distances to find transport and commuting in congested, dangerous transport. Thus, there is an urgent need to investigate the role of air pollutants in the different MC neighborhoods and their association with children's cognitive and behavior responses.

Epidemiological studies should be carried out to precisely determine the spatial distribution of air pollution health risks, followed by environmental protection measures and public health interventions. Research addressing low SES children's physiological regulatory capacities and cognition and developmental outcomes should also be carried out. To address the low SES children's detrimental responses to their physical and social environments (Jerrett et al., 2001; Morello-Frosch et al., 2002; Cicchetti et al., 2010, 2011; Rogosch et al., 2011; Prochaska et al., 2012; Sturge-Apple et al., 2012), efforts should be aimed to:

1) modify the current public school curricula to build executive function skills and cognitive reserves, which require trained supportive teachers and good quality school infrastructure;
2) provide access to free school lunch with balanced healthy diets; and
3) facilitate access to free good pediatric care, including mental health services.

Furthermore, if early childhood air pollution exposures related to SES disadvantage can increase the neurodevelopmental and neurodegenerative risk in the exposed child, then the need for interventions aimed at breaking the cycle of childhood poverty, poor food security, high unemployment, air pollution, and the negative health consequences becomes heightened.

We envisioned the protection of children to include cognitive interventions (Diamond and Lee, 2011), but it is important to remember that these approaches work if the children's emotional and social needs are also fulfilled. All efforts – nutritional, academic, extracurricular – are necessary, but insufficient if indeed the air children breath is not clean, and the environment is violent and stressful.

We need environmental justice for Mexico City exposed children. As Cureton (2011) stated, “*environmental injustice recognizes that economically disadvantaged groups are adversely affected by environmental hazards more than other groups*.” Low SES MC children urgently need a support system involving parents, teachers, the health system, and government initiatives to improve environmental health. Besides addressing short-term brain outcomes, we need to investigate the long effects of neuroinflammation and if we are facing an Alzheimer's/Parkinson's epidemic in 30 years. Comprehensive epidemiologic investigations of air pollutant components exposure in pediatric populations and social health outcomes, including measures of delinquent or criminal activity are also needed (Haynes et al., 2011). Childhood aggression and teen delinquency are increasing in Mexico City, establishing an early environmental health risk factor for violence prediction, and prevention (Liu, 2011) in populations at risk will be absolutely critical.

Unfortunately while we wait for governmental sectors to address these endemic issues, there are no coverings for our children's noses, nor for their lungs, hearts or vulnerable brains. The body of knowledge gleaned from rigorous air pollution studies should be taken seriously by those concerned with health policy and public health. The unfortunate combination of poverty and air pollution are causing serious adverse, and often irreversible, health outcomes in our children.
